# The complete chloroplast genome sequence of the biofuel plant Sacha Inchi, *Plukenetia volubilis*

**DOI:** 10.1080/23802359.2018.1437816

**Published:** 2018-03-09

**Authors:** Xiao-Di Hu, Bang-Zhen Pan, Qiantang Fu, Mao-Sheng Chen, Zeng-Fu Xu

**Affiliations:** a Key Laboratory of Tropical Plant Resources and Sustainable Use, Xishuangbanna Tropical Botanical Garden, Chinese Academy of Sciences, Menglun, Yunnan, China;; b College of Life Sciences, University of Chinese Academy of Sciences, Beijing, China

**Keywords:** *Plukenetia volubilis*, chloroplast genome, phylogenetic relationship

## Abstract

Sacha Inchi (*Plukenetia volubilis*) is a potential woody oil seed plant for producing healthy vegetable oil due to high content of α-linolenic acid in its seeds. In this study, we report the structure of the complete chloroplast genome of *P. volubilis* using high-throughput next-generation sequencing technology. The circular chloroplast genome is 161,733 bp in size, containing a pair of inverted repeat regions (IR) of 27,382 bp each, which were separated by a large single copy region (LSC) of 88,843 bp and a small single copy region (SSC) of 18,126 bp. The chloroplast genome harbors 135 genes, including 92 protein-coding genes, 35 tRNA genes and 8 rRNA genes. Based on the phylogenetic relationships between the chloroplast genome of *P. volubilis* and those of the other species, *P. volubilis* is most closely related to castor bean (*Ricinus communis*).

Sacha Inchi (*Plukenetia volubilis*), also known as Inca peanut, belongs to the genus *Plukenetia* of tribe Plukenetieae in the family Euphorbiaceae (Gillespie 2007). Sacha Inchi seeds have high content of α-linolenic acid, which is an essential fatty acid in our diet (Burdge 2006; Gutierrez et al. 2011; Chirinos et al. 2013). So far little genetic information on Sacha Inchi is reported. The chloroplast genome information was extensively applied in understanding plant genetic diversity and evolution (Ye et al. 2014). In this study, we assembled and annotated the chloroplast genome of *P. volubilis* using high-throughput next-generation sequencing technology. *P. volubilis* was introduced and cultivated to xishuangbanna of China in 2006 (Cai 2011), and young leaves were collected at the Xishuangbanna Tropical Botanical Garden, the Chinese Academy of Sciences, Menglun, Yunnan, China (21°54′N, 101°46′E, 580 m above sea level). Total genomic DNA was extracted with the CTAB method (Doyle 1986). According to the manufacturer’s manual (Illumina, San Diego, CA), the pair-end and long mate-pair libraries were constructed and then sequenced using Hiseq2000 platform. *De novo* assembly of chloroplast genome was performed using the SPAdes with the default parameters (Bankevich et al. 2012). The assembled contigs were aligned and ordered with the chloroplast genome of castor bean (*Ricinus communis*, GenBank accession number: NC_016736), whose genome is closest to that of Sacha Inchi. The plastome was annotated by Dual Organellar GenoMe Annotator (DOGMA) (Wyman et al. 2004), and the annotated genomic sequence was deposited in GenBank with the accession number MF062253.

The circular genome of *P. volubilis* is 161,733 bp in size, and comprises a pair of inverted repeat (IR) regions of 27,382 bp each, a large single-copy region (LSC) of 88,843 bp and a small single-copy region (SSC) of 18,126 bp. The chloroplast genome harbours 135 genes, including 92 protein-coding genes, 35 tRNA genes and 8 rRNA genes. The overall GC content of the chloroplast genome is 36.2% that is similar to other Euphorbiaceae chloroplast genomes Table 1, and those of the LSC and SSC regions are 33.7% and 30.5%, respectively. The size of Euphorbiaceae chloroplast genomes is in the range of 161–164 kbTable 1.

**Table 1.** Comparison of Euphorbiaceae chloroplast genomes reported in this study.

	*P. volubilis*	*R. communis*	*H. brasiliensis*	*M. esculenta*	*J. curcas*	*E. esula*
Size (bp)	161,733	163,161	161,191	161,453	163,856	160,512
Number of genes	135	131	129	128	129	132
GC content (%)	36	36	36	36	35	36

To determine the phylogenetic position of *P. volubilis*, a phylogenetic analysis was carried out among eight complete chloroplast genomes that are derived from *P. volubilis*, *Ricinus communis*, *Hevea brasiliensis*, *Manihot esculenta*, *Jatropha curcas*, *Euphorbia esula*, *Arabidopsis thaliana* and *Oryza sativa*. The chloroplast genome sequences of the other seven species except *P. volubilis* were downloaded from National Center for Biotechnology Information (NCBI, https://www.ncbi.nlm.nih.gov/). Alignment of sequences was performed using MAFFT (Katoh and Standley 2013). The maximum-likelihood analyses were accomplished using the program MEGA 7 (Kumar et al. 2016). The results showed that all six species of the family Euphorbiaceae formed a single cluster, and *P. volubilis* is closest to *R. communis* (Figure 1). The assembled chloroplast genome of *P. volubilis* reported in this paper will provide valuable information on the genetic diversity research of *P. volubilis*, and enrich the resources of chloroplast genomes in plants.

**Figure 1.**
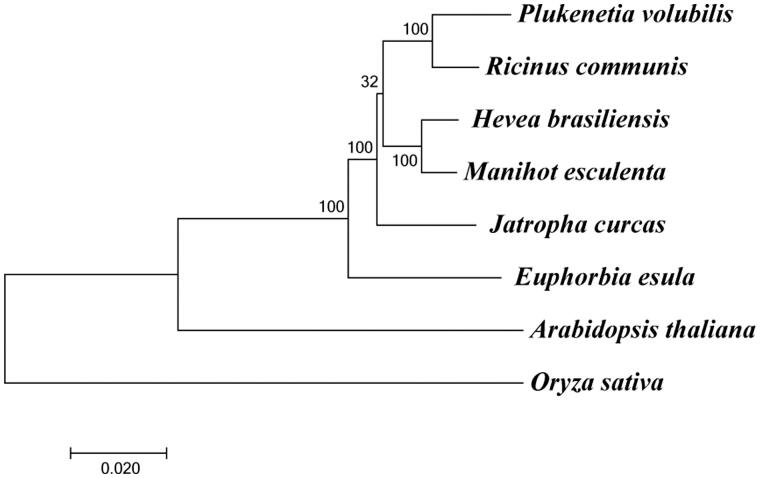
Maximum-likelihood phylogenetic tree of eight species. These species include *Plukenetia volubilis* (MF062253), *Ricinus communis* (NC_016736.1), *Hevea brasiliensis* (NC_015308.1), *Manihot esculenta* (NC_010433.1), *Jatropha curcas* (NC_012224.1), *Euphorbia esula* (NC_033910.1), *Arabidopsis thaliana* (NC_000932.1), and *Oryza sativa* (NC_027678.1). The numbers at the nodes are bootstrap values with 1000 replicates.
